# Discovery and systematic assessment of early biomarkers that predict progression to severe COVID-19 disease

**DOI:** 10.1038/s43856-023-00283-z

**Published:** 2023-04-12

**Authors:** Katrin Hufnagel, Anahita Fathi, Nadine Stroh, Marco Klein, Florian Skwirblies, Ramy Girgis, Christine Dahlke, Jörg D. Hoheisel, Camille Lowy, Ronny Schmidt, Anne Griesbeck, Uta Merle, Marylyn M. Addo, Christoph Schröder

**Affiliations:** 1 Sciomics GmbH, Neckargemünd, Baden-Württemberg Germany; 2 University Medical Center Hamburg-Eppendorf, Institute for Infection Research and Vaccine Development (IIRVD), Hamburg, Germany; 3 Bernhard-Nocht-Institute for Tropical Medicine, Department for Clinical Immunology of Infectious Diseases, Hamburg, Germany; 4 German Center for Infection Research, partner site Hamburg-Lübeck-Borstel-Riems, Hamburg, Germany; 5 University Medical Center Hamburg-Eppendorf, First Department of Medicine, Division of Infectious Diseases, Hamburg, Germany; 6 Division of Functional Genome Analysis, German Cancer Research Center (DKFZ), Heidelberg, Baden-Württemberg Germany; 7 Department of Internal Medicine IV, University Hospital Heidelberg, Heidelberg, Germany

**Keywords:** SARS-CoV-2, Biomarkers, Viral infection, Proteomics

## Abstract

**Background:**

The clinical course of COVID-19 patients ranges from asymptomatic infection, via mild and moderate illness, to severe disease and even fatal outcome. Biomarkers which enable an early prediction of the severity of COVID-19 progression, would be enormously beneficial to guide patient care and early intervention prior to hospitalization.

**Methods:**

Here we describe the identification of plasma protein biomarkers using an antibody microarray-based approach in order to predict a severe cause of a COVID-19 disease already in an early phase of SARS-CoV-2 infection. To this end, plasma samples from two independent cohorts were analyzed by antibody microarrays targeting up to 998 different proteins.

**Results:**

In total, we identified 11 promising protein biomarker candidates to predict disease severity during an early phase of COVID-19 infection coherently in both analyzed cohorts. A set of four (S100A8/A9, TSP1, FINC, IFNL1), and two sets of three proteins (S100A8/A9, TSP1, ERBB2 and S100A8/A9, TSP1, IFNL1) were selected using machine learning as multimarker panels with sufficient accuracy for the implementation in a prognostic test.

**Conclusions:**

Using these biomarkers, patients at high risk of developing a severe or critical disease may be selected for treatment with specialized therapeutic options such as neutralizing antibodies or antivirals. Early therapy through early stratification may not only have a positive impact on the outcome of individual COVID-19 patients but could additionally prevent hospitals from being overwhelmed in potential future pandemic situations.

## Plain language summary

We aimed to identify components of the blood present during the early phase of SARS-CoV-2 infection that distinguish people who are likely to develop severe symptoms of COVID-19. Blood from people who later developed a mild or moderate course of disease were compared to blood from people who later had a severe or critical course of disease. Here, we identified a combination of three proteins that were present in the blood of patients with COVID-19 who later developed a severe or critical disease. Identifying the presence of these proteins in patients at an early stage of infection could enable physicians to treat these patients early on to avoid progression of the disease.

## Abstract (web summary)

Hufnagel, Fathi et al. use an antibody microarray-based approach to identify plasma protein biomarkers present during the early phases of SARS CoV-2 infection that distinguish people who will go on to develop severe COVID-19. A multimarker panel of proteins enables people at high risk of developing a severe or critical disease course to be identified.

## Introduction

The outbreak of the coronavirus disease 2019 (COVID-19), caused by severe acute respiratory syndrome coronavirus 2 (SARS-CoV-2), was officially declared a global pandemic on March 11, 2020 by the World Health Organization (WHO). The first infection was reported by the end of December 2019 and almost two years later, the WHO reported more than 260 million confirmed cases of COVID-19, including 5.2 million deaths^1^. The clinical course of the disease can range from asymptomatic infection, mild and moderate disease, to severe and even critical disease courses with a case-fatality rate of up to 30% in patients older than 85 years^2,3^. The underlying factors causing the remarkable clinical variation of disease course are subject to intense investigation. While it is well-established that certain predisposing conditions, above all high age and, secondly, certain comorbidities^4^, are associated with an elevated risk for severe COVID-19, clinical outcomes still vary widely within these groups and it is not well understood which factors lead to an adverse outcome^5^. The identification of sensitive and specific prognostic biomarkers to predict the clinical trajectory is, therefore, crucial to guide clinical care and early intervention to ideally avoid the necessity of critical care. Several studies have described differential blood levels of various pro- or anti-inflammatory cytokines, chemokines and other proteins in sera of severely ill COVID-19 patients and suggested their use as prognostic markers for the outcome of SARS-CoV-2 infections or even as potential therapeutic targets^6–9^. However, most previous studies only report the analysis of a few or a single potential biomarker(s), which often lack accuracy for a clinical application. While COVID-19 vaccines have shown to be remarkably protective against severe disease, insufficient global vaccine coverage, waning immunity, and the emergence of new variants remain challenges to prevention and underline the need for effective COVID-19 treatment strategies. Immune-escape variants such as the omicron variant, furthermore, render the majority of monoclonal antibodies currently used as treatment or post-exposure prophylaxis in a wide array of patients at risk for severe COVID-19 ineffective. Due to the shortage of effective therapies against COVID-19, it is essential to select sensitive and specific biomarkers that allow in the first days of the disease and prior to an aggravation of the disease to identify those patients who are at the highest risk of developing a severe or critical course of disease and circumvent such a progression by an early treatment.

Here, we describe a study in which plasma levels of potential biomarkers were measured on antibody microarrays at an early phase of infection in COVID-19 patients which exhibited afterwards either a mild to moderate or severe to critical course of disease. Antibody microarrays are an analysis platform using antibodies immobilized on a glass slide. Patient samples were incubated on individual arrays with 1425 antibodies directed against 998 proteins. We were able to detect significantly differential plasma levels for 11 biomarkers when comparing mild to moderate and severe to critical patients during an early phase of infection in two independent cohorts. A machine learning approach revealed a combination of four biomarkers with the highest sensitivity and specificity. Individual markers could be validated by commercial ELISA assays. As the discovery approach was already completely immune-based, the biomarker combinations have the potential to be successfully translated into a clinical diagnostic tool.

## Materials & methods

### Study design

After obtaining written informed consent, whole blood samples from SARS-CoV-2 infected individuals, who had been diagnosed by RT-PCR using a nasopharyngeal swab, were collected at University Medical Center Hamburg-Eppendorf (1^st^ cohort) and the Department of Gastroenterology and Infectious Diseases of University Hospital Heidelberg (2^nd^ cohort) at different timepoints after diagnosis. Both studies were conducted according to the ethical requirements established by the Declaration of Helsinki. The 1^st^ study was approved by local Ethics Committee of the Hamburg Medical Association (ethic consent number PV7298), while the 2^nd^ study was approved by the local Ethics Committee of the Medical Faculty of Heidelberg University Hospital (ethic consent number S-148/2020).

All samples from the 1^st^ cohort were classified into three phases of infection based on the onset of symptoms: acute (up to nine days after symptom onset), intermediate (10–21 days after symptom onset), and late (more than 21 days after symptom onset). All samples analyzed from the 2^nd^ cohort were collected during the acute phase of infection.

Disease severity was classified according to the definition of the WHO-China Joint Mission on Coronavirus Disease^10^, which is commonly utilized as a classification, among others by the Infectious Disease Society of America (IDSA) and the Robert Koch Institute in Germany. Briefly, these categories are defined as: mild = no sign of pneumonia; moderate = radiologic evidence of pneumonia, blood oxygen saturation >93%, no supplemental oxygen therapy; severe = supplemental oxygen therapy necessary; critical = respiratory or multi-organ failure. Patient “a” of the 1^st^ cohort represented a borderline case, who presented a poor condition with high fever, pneumonia and required hospitalization, and whose disease course was judged as clinically severe as described earlier^11^.

### Microarray analysis

Sample labelling and incubation were performed as previously described in detail^12–14^. In brief, plasma samples from COVID-19 patients were labeled at an adjusted protein concentration for two hours with the fluorescent dye scioDye 2 (Sciomics, Neckargemünd, Germany). As reference sample, a pool composed of all samples included in each sample cohort was used and labeled with a second dye (scioDye 1). After two hours, the labeling reaction was stopped, and the buffer exchanged to Phosphate-Buffered Saline (PBS). For improved assay robustness and differentiation power, each sample was competitively incubated together with a common reference sample on one microarray slide in a reference-based dual-color approach as described in detail before^15^.

The 53 samples of the 1^st^ cohort were analyzed on 53 scioCD antibody microarrays (Sciomics) targeting 351 different proteins by 517 antibodies (Supplementary Data 1). The 94 samples of the 2^nd^ cohort were analyzed on antibody microarrays (Sciomics) targeting 998 different proteins by 1425 antibodies (Supplementary Data 2), each in four replicates. All proteins that were analyzed in the 1^st^ cohort were also analyzed in the 2^nd^ cohort. Array surfaces were blocked with scioBlock (Sciomics) on a Hybridization Station 4800 PRO (Tecan, Grödig, Austria) and the samples were subsequently incubated competitively with the reference sample using a dual-color approach. After incubation for three hours, the slides were thoroughly washed with 1x PBSTT (Phosphate-buffered saline containing Tween and Triton), rinsed with 0.1x PBS as well as with water and subsequently dried with nitrogen.

### Data acquisition and analysis

Slide scanning was conducted using a Powerscanner (Tecan, GmbH, Grödig, Austria) with constant instrument laser power and photomultiplier settings. Spot segmentation was performed with GenePix Pro 6.0 (Molecular Devices, Union City, USA). The acquired raw data were analyzed using the linear models for microarray data (limma 3.42.2) package of R-Bioconductor after uploading the median signal intensities. For normalization, a specialized invariant Lowess method was applied^16^. For both cohorts, a multi-factorial linear model was fitted using limma, integrating patient age, sex and comorbidities next to the main factor. For the first cohort, this main factor combined disease severity and phase, while for the 2^nd^ cohort, the main factor consisted only of disease severity as the cohort exclusively consisted of acute phase samples. The factors age and comorbidities were defined in a binary manner, detailing age equal to or higher than 60 and presence of at least one comorbidity, respectively. Differences between sample groups were calculated based on the fitted group means generated by the linear model and are presented as log-fold changes (logFC) calculated for the basis 2.

### Statistics and reproducibility

*p* values were obtained from moderated two-sided *t*-tests based on empirical Bayes moderation. All presented *p* values were adjusted for multiple testing by controlling the false discovery rate according to Benjamini and Hochberg. Proteins were defined as differentially abundant for |logFC | > 0.5 and an adjusted *p* value <0.05. Descriptive data is depicted by range, mean and standard deviation (SD).

The 1^st^ cohort comprised a total of 53 samples, including 17 acute phase samples (14 mild/moderate, 3 severe/critical), 20 intermediate phase samples (13 mild/moderate, 7 severe/critical) and 16 late phase samples (10 mild/moderate, 6 severe/critical), while the 2^nd^ cohort consisted of a total of 94 samples, including 47 matched pairs of mild/moderate and severe/critical samples.

Signal intensity measurements for each antibody and sample were performed with four technical replicates spaced out across each microarray. The average estimated inter-replicate correlation was included into the statistical analysis.

### Machine learning

Potential biomarker combinations were determined via linear support vector machine (linSVM) models using the SVC implementation of scikit-learn package (0.24.2) in python (3.8.10)^17^.

Before the machine learning process, a pre-selection of single markers was performed based on two criteria. Firstly, the 11 markers which displayed discriminative power based on logFC and *p* values obtained from the linear models of both cohorts were included. Secondly, the top 10 markers according to a preliminary linSVM coefficient criterion as detailed in Supplementary Table 1 were included for the pre-selection, resulting in a total of 21 different antibodies detecting 20 protein biomarker candidates.

From this marker set, all possible combinations of two, three and four biomarkers were tested to differentiate between patients with a critical/severe or mild/moderate course of the disease. Due to the limited size of the data set for machine learning applications, the performance of a linSVM model for a marker combination was gauged using ROC AUC estimation in a Leave-One-Out (LOO) format^18^. For the calculation of ROC curves, instead of binary predictions, class probabilities for the class “critical-severe”, as generated from the predict_proba method of the SVC implementation, were predicted for each LOO split. This resulted in one set of predictions per biomarker combination, which was subsequently compared with the samples’ real labels to calculate the ROC AUC. In order to keep the trained models similar across the LOO folds, models were trained with a static cost parameter C = 1. The robustness of the linSVM models across the LOO folds was assessed by the coefficient of variation (CV) of the linear coefficients fitted for each fold. For the combination S100A8/A9 and CRP the CV was 12.6%, for all other individual markers and combination the CVs were in the range of 2–6% indicating robust models within the LOO process.

### Validation of biomarkers

Concentrations of S100A8/A9 in human plasma samples were measured using the Human S100A8/S100A9 Heterodimer DuoSet Enzyme-linked Immunosorbent Assay (ELISA) (R&D Systems) according to the manufacturer’s protocol. Plasma samples were diluted 1:1000 in PBS containing 1% BSA in order to allow a measurement within the detection limits of the ELISA.

CRP was analyzed in plasma samples at the accredited central laboratory of the Heidelberg university hospital on a Siemens ADVIA Chemistry XPT System (CRP reagent kit 00829585) according to the manufacturer’s instructions.

## Results

### Study population 1^st^ cohort

**Fig. 1:**
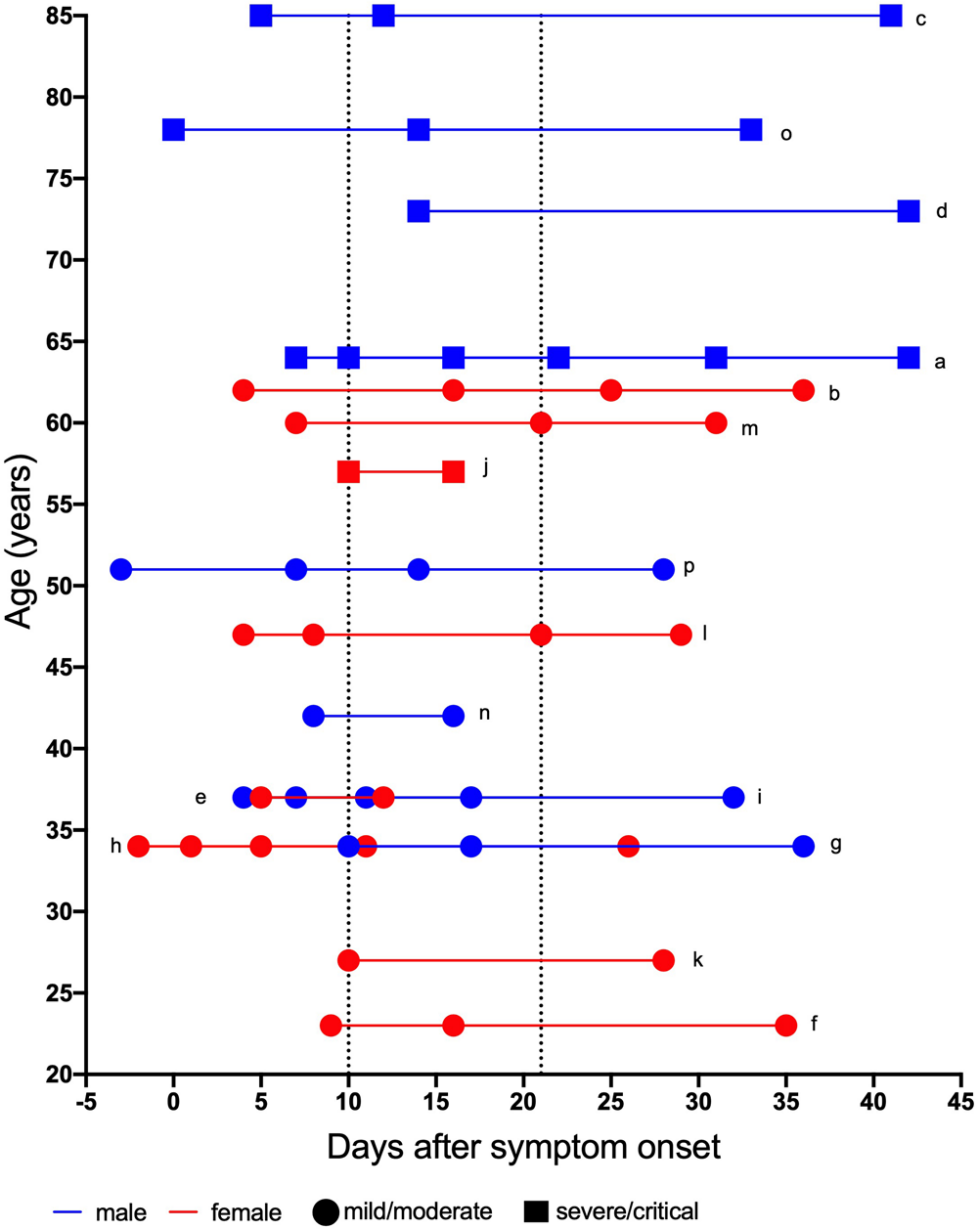
Patient and sample characteristics of the 1^st^ cohort. Characteristics (age, sex) of individuals included in this study and timepoints of sample collection after symptom onset. Letters a–p indicate the individual patients. Male patients are depicted in blue, female patients in red. Circles represent samples of mild or moderate (MM) patients, squares those of critical or severe (CS) patients. Acute phase: 14 MM and 3 CS samples; intermediate phase: 13 MM and 7 CS samples; late phase: 10 and 6 samples. Dotted vertical lines indicate 10 days and 21 days after onset of symptoms.

We analyzed 53 plasma samples collected longitudinally from 16 COVID-19 patients. The cohort included eight men and women, respectively, aged between 23 and 85 years (mean: 47 years, SD: 18 years). Since it has been observed that the course of COVID-19 can deteriorate several days after the onset of first symptoms, we decided to divide the disease course into three periods based on days since the onset of first symptoms: an acute (<10 days) an intermediate (between 10 and 21 days) and a convalescent/late stage (>21 days), for which we included 17, 20 and 16 samples, respectively (see Fig. 1 for patient/sample characteristics). In all individuals, the day of first symptom onset was between February 25^th^ and April 30^th^, 2020, and the cohort, therefore, mirrors an early time of the pandemic, when specific therapies against COVID-19 had not been established. Immuno-modulating or -suppressive conditions and therapies were recorded for all participants to be able to assess whether these conditions/treatments influenced our findings. Of the 16 individuals, 2 were pregnant (patients e and h), one individual received 7.5 mg prednisolone (patient g), another one 6 mg dexamethasone daily due to underlying comorbidities (patient o) and one had received tocilizumab as a compassionate use treatment of COVID-19 (j). The remaining 11/16 patients did not have any immuno-modulating or -suppressive condition and did not receive any immunosuppressive therapy.

### Discovery of disease severity markers within the 1^st^ cohort

In order to identify differentially abundant proteins, human plasma samples from COVID-19 patients with either a mild or moderate (MM) or a critical or severe (CS) disease course from different disease phases (acute, intermediate, late) were analyzed on antibody microarrays targeting 351 different proteins via 517 antibodies.

**Fig. 2:**
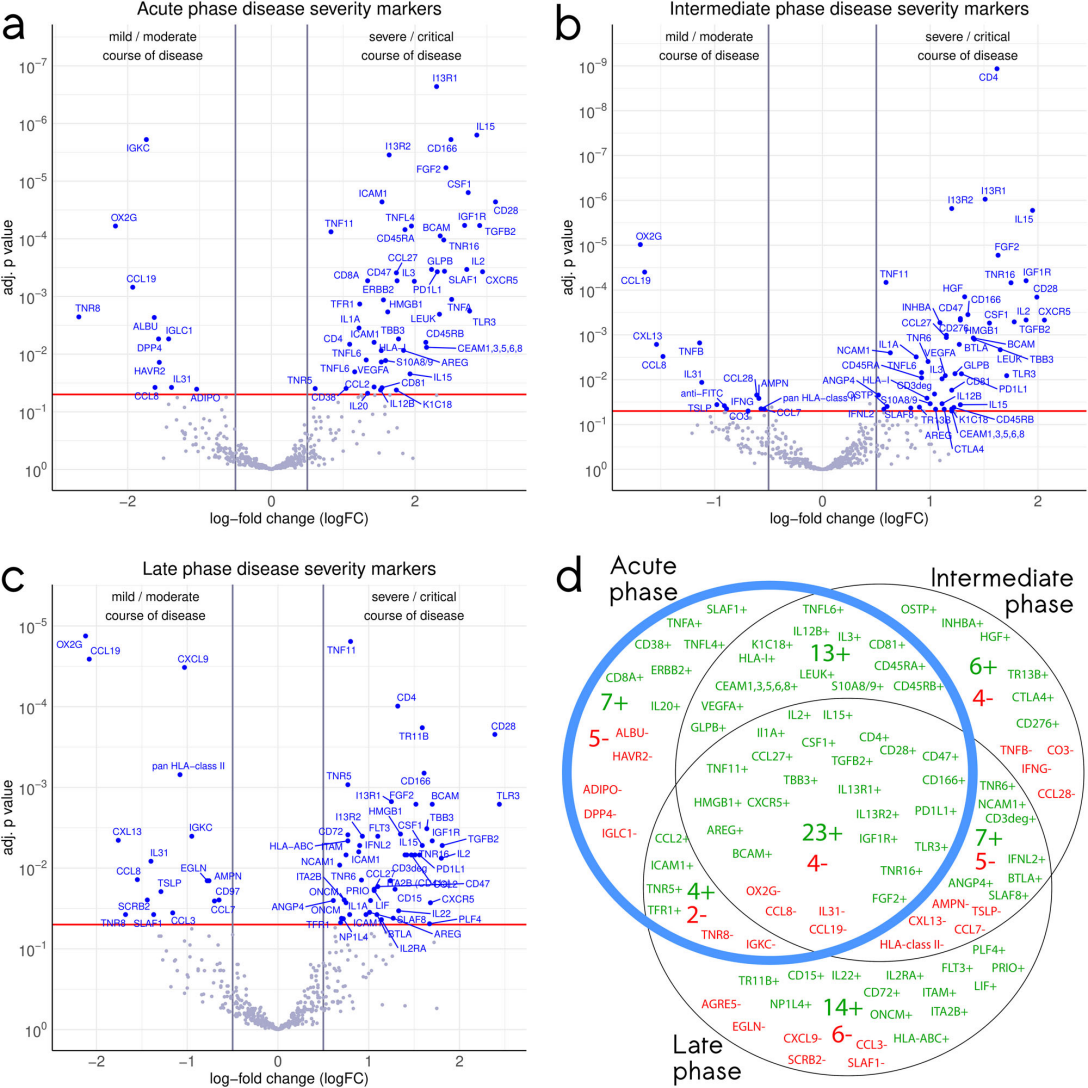
Venn diagram and volcano plots illustrating the number, degree and significance of differential protein expression in the 1^st^ cohort. The volcano plots visualize the p values (adjusted for multiple testing) and corresponding log-fold changes (logFC) of the identified protein biomarker candidates. A significance level of adj. *p* value = 0.05 is indicated as a horizontal red line. Absolute logFC cutoffs of |logFC | > 0.5 are indicated as vertical lines. 53 plasma samples from 16 COVID-19 patients were analyzed and divided into an acute (**a**) an intermediate (**b**) and a late stage (**c**), for which we included 18, 20 and 16 samples, respectively. Proteins with a positive logFC had a higher abundance in CS samples, proteins with a negative value in MM samples. **d**: Venn diagram listing the differential proteins and their numbers in the respective phases. Green numbers and protein IDs indicate proteins more abundant in CS patients and red numbers and IDs proteins with higher abundance in MM patients.

100 proteins showed a significant differential abundance in CS compared to MM COVID-19 patients, from which 74 were higher and 26 lower abundant, while 417 antibodies did not show a significant difference. When classifying the samples additionally into different phases, we recorded 58 differentially abundant proteins in the acute phase (Fig. 2a), 62 in the intermediate (Fig. 2b) and 65 in the late phase (Fig. 2c). There is a broad overlap between differentially abundant proteins in CS compared to MM patients in all three phases of infections, especially between acute and intermediate phase (Fig. 2d). However, there are proteins that only show differential abundance during a specific phase of infection.

The 100 differentially abundant proteins were identified based on fitted group means generated by a linear model and thereby adjusting means for age, sex and comorbidities. This allows the identification of biomarkers associated specifically with severity and phase of disease and minimizes the chance of identifying biomarkers associated with any of these confounders.

**Fig. 3:**
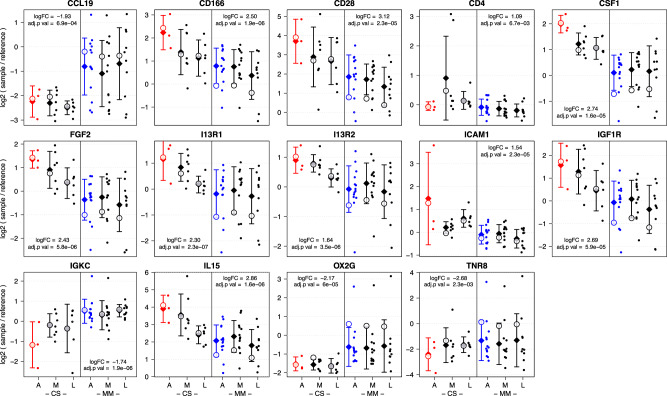
Stripcharts representing individual array values for all proteins selected as top candidates in the 1^st^ cohort. Each protein is measured by four replicate spots per array and is represented by their mean. The y-axis illustrates the log2 ratio of the individual samples and a reference sample while the x-axis is divided based on clinical course of disease of the patient (CS and MM) as well as phase of infection. 53 plasma samples from 16 COVID-19 patients were analyzed and divided into an acute (A) an intermediate (M) and a late stage (L), for which we included 18, 20 and 16 samples, respectively. Acute CS and MM samples are highlighted in red and blue respectively. Diamonds indicate arithmetic sample group means. Whiskers indicate one standard deviation, calculated based on arithmetic means. Empty circles indicate the group coefficients fitted by the linear model with additional factors sex, age and comorbidities, which were used for logFC and *p* value calculation.

All analyzed proteins with their respective logFC and *p* values in CS and MM patients in the three disease phases are listed in Supplementary Data 1. For each protein, the Uniprot Entry-Name and ID is listed together with the logFC and adjusted *p* values as illustrated in Fig. 2. Out of these 100 proteins, 14 top candidates were identified discriminating acute CS and acute MM patients at a |logFC | > 1.0 and a significance level of adj.*p* < 0.05 (Fig. 3). Additionally, CD4 exhibited a high significance in intermediate phase CS patients compared to intermediate phase MM patients (Fig. 2b). Most of the selected top candidates were able to discriminate between patients with a CS and MM course of disease in the acute phase within this sample set (Fig. 3). Differences in protein levels observed in the acute phase vanished for a high number of targets within the course of disease, especially for proteins higher abundant in CS (Fig. 3). A certain heterogeneity was observed in the patient cohort in this analysis and is illustrated by the following examples. The stripchart for CD4 illustrates that this difference is mainly driven by two samples which both belong to the same patient. Similarly, differences in ICAM1 abundance might be strongly influenced by a single CS sample with extremely high ICAM1 levels, while differences in TNR8 levels can be explained by low levels in one CS sample and additionally high levels in all samples from one MM patient in all three phases of the infection. These examples highlight that some of the differences observed within the comparatively small 1^st^ cohort might be driven by certain patients and therefore only be present in individuals or a subpopulation. To validate the identified targets and select biomarkers, that are not only present in individuals or subpopulations, a second larger and more heterogeneous cohort was analyzed.

### Study population 2^nd^ cohort – predicting a severe COVID-19 disease

In order to assess specifically the potential to predict a severe COVID-19 disease already at an early stage of the disease, a second cohort was included in the study. The cohort consisted of 94 plasma samples from COVID-19 patients during the acute phase of disease (<10 days after the onset of first symptoms). At this stage of the disease the patients had no severe symptoms and were not in need for intensive care or hospitalisations. From these 94 patients, 47 patients later had a critical or severe course of disease, while 47 age and sex matched patients had a mild to moderate disease course only. The characteristics of these samples are summarized in Table 1. All patients survived the infection and did not receive COVID-19 specific medication prior to sample collection.

**Table 1:** Sample characteristics of the 2^nd^ cohort.

	critical/severe	mild/moderate
Number of samples	47	47
Mean age (SD)	60.2 (13.4)	60.2 (12.7)
Age range	23–80	30–82
male/female	66%/34%	66%/34%
mean days after onset of symptoms (SD)	6.3 (2.1)	6.0 (2.3)

### Analysis of markers for prediction of a severe course of a COVID-19 disease within the 2nd cohort

To identify additional protein markers and verify the findings from the initial cohort, the 94 plasma samples from the 2^nd^ cohort were analyzed on antibody microarrays targeting 998 different proteins by 1425 antibodies.

**Fig. 4:**
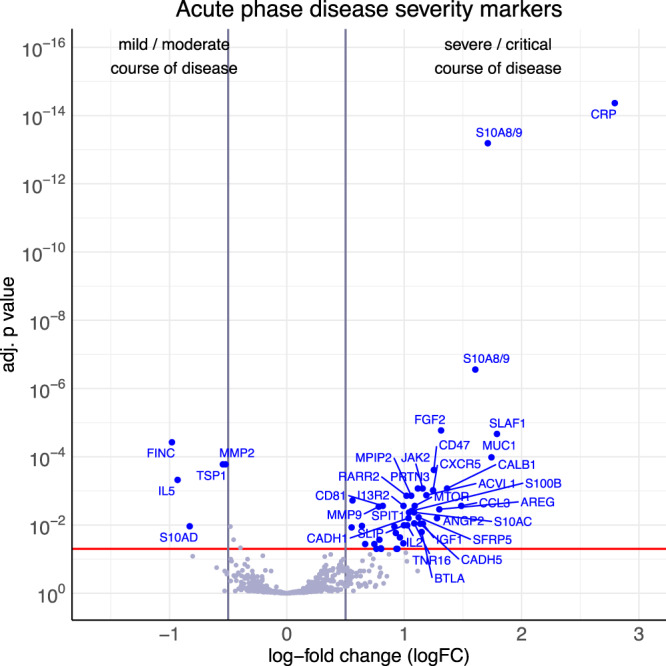
Protein biomarker candidates from the 2^nd^ cohort to predict a severe or critical disease in acute phase. Plasma samples from 94 COVID-19 patients during the acute phase of disease were analyzed on antibody microarrays to identify differentially abundant proteins between patients with either a mild or moderate (MM, *n* = 47) or a critical or severe (CS, *n* = 47) disease course. The volcano plot visualizes the *p* values and corresponding log-fold changes (logFC). A significance level of adj. *p* value = 0.05 is indicated as a horizontal red line. Absolute logFC cutoffs of |logFC | > 0.5 are indicated as vertical lines. Proteins with a positive logFC had a higher abundance in CS samples, proteins with a negative value in MM samples. Two different antibodies against S10A8/A9 were included on the microarray, with both antibodies showing significant differences between CS and MM samples.

In this cohort, 51 proteins were differentially abundant in patients with a CS or MM course of the disease, from which 46 were higher abundant and five lower abundant, while 947 proteins did not show a significant difference (Fig. 4). Proteins positively associated with a severe course of the COVID-19 disease (positive logFC), such as CRP, S100A8/A9 (detected by two different antibodies), FGF2 and SLAF1, while FINC, TSP1, MMP2, IL5 and S10AD (negative logFC) were less abundant in plasma of patients with a severe or critical course of the disease. All analyzed proteins with their respective abundance in CS and MM patients are listed in Supplementary Data 2. For each protein, the Uniprot Entry-Name and ID is listed together with the logFC and adjusted *p* values.

For a mechanistic understanding of proteins altered in a CS course of the disease, the differential proteins were subjected to a protein interaction analysis using the STRING database (Supplementary Fig. 1)^19^. The interaction pointed specifically to a regulation of many S100 family proteins such as S100A8/A9 and S100B in combination with HMBG1. Furthermore, the Janus kinase/signal transducer and activator of transcription (JAK/STAT) pathway can be seen as one of the central axes in Supplementary Fig. 1.

### Biomarkers predictive for disease severity markers in both study cohorts

**Fig. 5:**
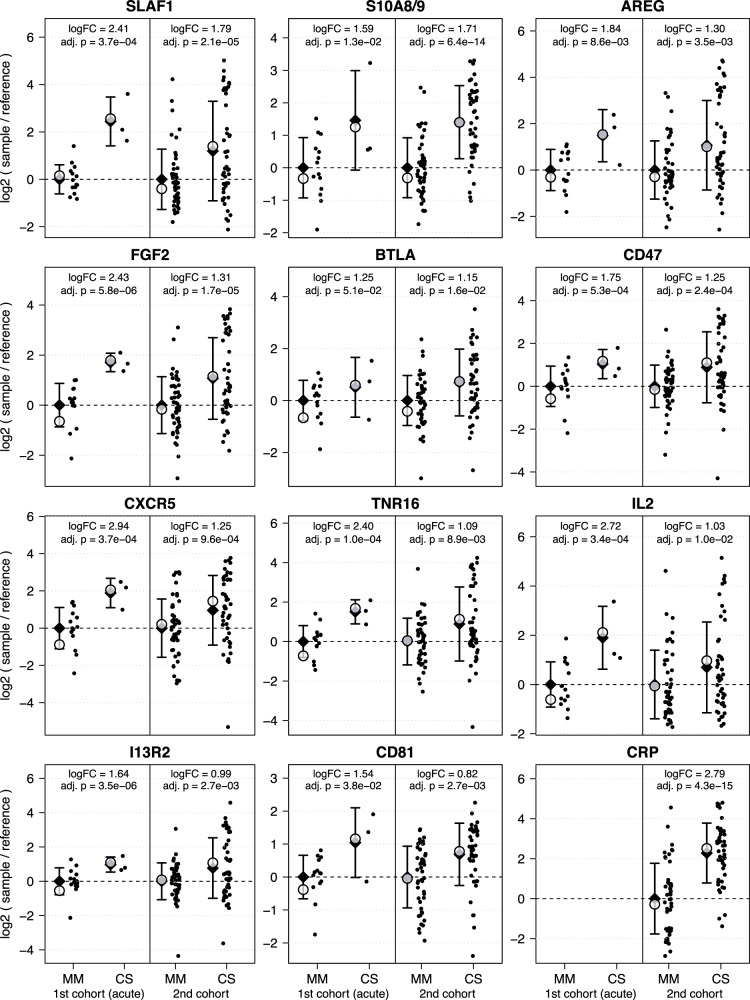
Biomarkers to predict a severe / critical disease in both study cohorts. The y-axis illustrates log2(sample / reference) values after subtracting the group mean of the respective MM samples per cohort/protein, thus setting the mean value of MM samples as a baseline. Within the 1^st^ cohort 18 samples (MM = 17; CS = 3) were analyzed, while 94 sample (MM = 47; CS = 47) were analyzed within the 2^nd^ cohort. The x-axis is divided based on clinical course of disease of the patient (CS and MM). Only acute CS and MM samples are shown. Diamonds indicate arithmetic sample group means. Whiskers indicate one standard deviation, calculated based on the arithmetic means. Empty circles indicate the group coefficients fitted by the linear model with additional factors sex, age and comorbidities, which were used for logFC and *p* value calculation.

From the 14 top candidates identified within the 1^st^ cohort, the two proteins FGF2 and I13R2 were also differentially abundant in the 2^nd^ cohort at a significance cutoff of adj.*p*-value <0.05. In total, eleven biomarkers were identified as predictive biomarkers for a severe COVID-19 disease in both study cohorts (Fig. 5, Table 2).

**Table 2:** Most important identified biomarkers within at least one of both cohorts.

	Target	Uniprot Name	Uniprot ID	1^st^ cohort		2^nd^ cohort	
				logFC	adj.*p*-val	logFC	adj.*p*-val
common targets	S100A8/A9	S10A8_HUMAN/S10A9_HUMAN	P05109/P06702	1.59	0.013	1.71	6.4 × 10^−14^
	FGF2	FGF2_HUMAN	P09038	2.43	5.8 × 10^−6^	1.31	1.7 × 10^−5^
	SLAF1	SLAF1_HUMAN	Q13291	2.41	3.7 × 10^−4^	1.79	2.1 × 10^−5^
	CD47	CD47_HUMAN	Q08722	1.75	5.3 × 10^−4^	1.25	2.4 × 10^−4^
	CXCR5	CXCR5_HUMAN	P32302	2.94	3.7 × 10^−4^	1.25	9.6 × 10^−4^
	I13R2	I13R2_HUMAN	Q14627	1.64	3.5 × 10^−6^	0.99	2.7 × 10^−3^
	CD81	CD81_HUMAN	P01889	1.54	0.038	0.82	2.7 × 10^−3^
	AREG	AREG_HUMAN	P31997	1.84	8.6 × 10^−3^	1.30	3.5 × 10^−3^
	TNR16	TNR16_HUMAN	P08138	2.40	1.0 × 10^−4^	1.09	8.9 × 10^−3^
	IL2	IL2_HUMAN	P60568	2.72	3.4 × 10^−4^	1.03	0.010
	BTLA	BTLA_HUMAN	Q7Z6A9	1.27*	1.6 × 10^−3^*	1.15	0.016
important targets 1st cohort	I13R1	I13R1_HUMAN	P78552	2.30	2.3 × 10^−7^	ns	ns
	IL15	IL15_HUMAN	P40933	2.86	1.6 × 10^−6^	ns	ns
	CD166	CD166_HUMAN	Q13740	2.50	1.9 × 10^−6^	ns	ns
	IGKC	IGKC_HUMAN	P01834	−1.74	1.9 × 10^−6^	ns	ns
	CSF1	CSF_HUMAN	P09603	2.74	1.6 × 10^−5^	ns	ns
	CD28	CD28_HUMAN	P10747	3.12	2.3 × 10^−5^	ns	ns
	IGF1R	IGF1R_HUMAN	P08069	2.69	5.9 × 10^−5^	ns	ns
	OX2G	OX2G_HUMAN	P41217	−2.17	6.0 × 10^−5^	ns	ns
	CCL19	CCL19_HUMAN	Q99731	−1.93	6.9 × 10^−4^	ns	ns
important targets 2nd cohort	CRP	CRP_HUMAN	P02741	na	na	2.79	4.3 × 10^-15^
	FINC	FINC_HUMAN	P02751	na	na	-0.98	3.7 × 10^-5^
	TSP1	TSP1_HUMAN	P07996	na	na	-0.55	1.7 × 10^-4^
	MUC1	MUC1_HUMAN	P15941	ns	ns	1.74	1.0 × 10^-4^
	CALB1	CALB1_HUMAN	P05937	na	na	1.37	8.5 × 10^-4^
	CCL3	CCL3_HUMAN	P10147	ns	ns	1.49	2.7 × 10^-3^
	ANGP2	ANGP2_HUMAN	O15123	na	na	1.28	6.3 × 10^-3^

^*^BTLA logFC and *p*-values (*p*-val.) for the 1^st^ cohort are from the intermediate phase of infection.

*ns* not significant.

*na* not available; target was not analyzed in this cohort.

For the analysis of the 2^nd^ cohort, microarrays targeting a broader range of proteins were used in order to assess additional biomarker candidates such as CRP. All biomarkers analyzed within the 1^st^ cohort were also included in the analysis of the 2^nd^ cohort. Within this set, we identified the following five additional biomarkers as top candidates with a |logFC | > 1.0 and a significance level of adj.*p* < 0.05: CRP, FINC, TSP1, CALB1 and ANGP2. In addition, the two biomarkers MUC1 and CCL3 reached the significance threshold in the 2^nd^ larger cohort only.

### ELISA reproduces findings of antibody array-based discovery

**Fig. 6:**
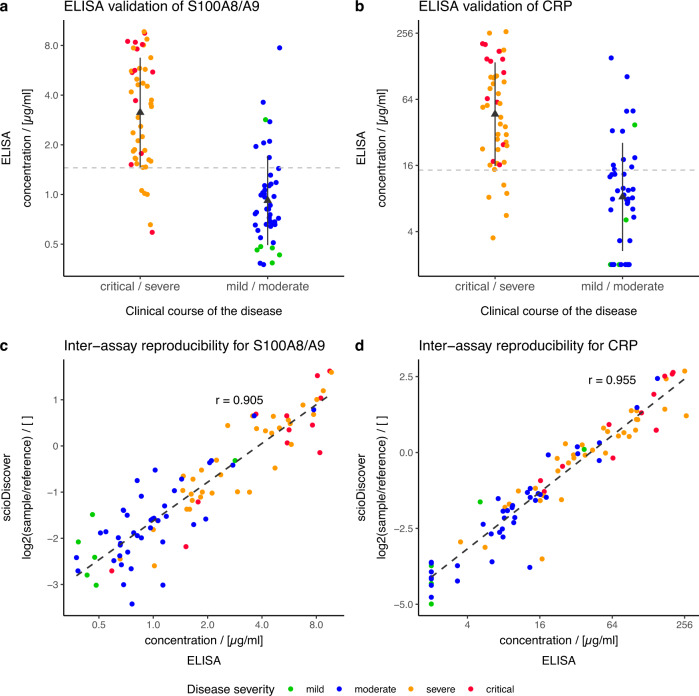
Validation of microarray data. Plasma samples from 94 COVID-19 patients during the acute phase of disease (2^nd^ cohort) were analyzed by ELISA, to validate differentially abundant proteins between patients with either a mild or moderate (MM, *n* = 47) or a critical or severe (CS, *n* = 47) disease course. Stripcharts representing individual S100A8/A9 (**a**) and CRP (**b**) ELISA measurements. The y-axis displays the log_2_ of the measured protein concentration while the x-axis is divided based on the later clinical course of disease of the patient (CS and MM). Triangles and whiskers indicate means and one standard deviation of the sample groups with critical/severe or mild/moderate course of the disease respectively. Possible cut-offs with a sensitivity of 89% are indicated by dotted grey lines. **c**, **d** Scatter plots demonstrate a high correlation between discovery antibody microarray data (y-axis) and ELISA validation (x-axis) with Pearson’s *r* > 0.9.

To prove transferability of our findings to other assay platforms, the array data for CRP of the larger study cohort were compared with clinical CRP data obtained at sample collection. In addition, the biomarker candidate S100A8/A9 exhibiting the second strongest discriminative power was chosen for such inter-assay comparison using a commercially available ELISA kit. Antibody array data and ELISA data exhibited a Pearson correlation coefficient of 0.905 for S100A8/A (Fig. 6c) and of 0.955 for CRP (Fig. 6d).

Also, in terms of discrimination power, the performance of the antibody array platform could be reproduced by ELISA. By applying a cutoff of 1.45 µg/ml for S100A8/A9 measured by ELISA, a specificity of 83% and a sensitivity of 89% were achieved. A cut off of 14.5 µg/ml for CRP measured by ELISA resulted in a specificity of 73% and a sensitivity of 89%.

### Biomarker combinations selected via machine learning for accurate disease severity prediction

**Fig. 7:**
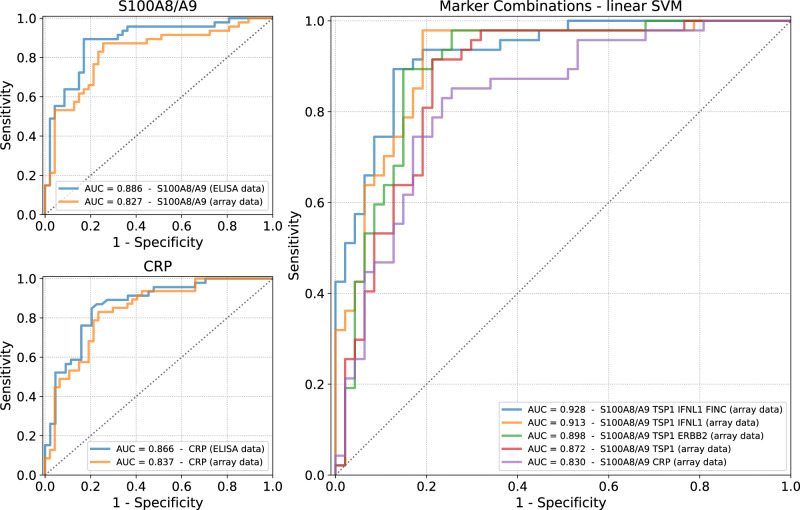
Estimation of diagnostic accuracy for disease severity prediction. For the individual protein biomarkers S100A8/A9 and CRP, ROC curves of ELISA data as well as coherent multiplex antibody array data were aligned. Marker combinations of 2–4 proteins were selected from linSVM models, which outperform the individual biomarker candidates and exhibit a high accuracy with an AUC of up to 0.928. Area under the ROC curve (AUC) is presented for each biomarker or combination.

In order to assess the diagnostic accuracy of the two top protein biomarker candidates, receiver operating characteristic (ROC) curves were generated for both the antibody array derived data and the ELISA data. For CRP, the area under the curve (AUC) was 0.837 based on the antibody array data and 0.866 for the ELISA data (Fig. 7). The biomarker S100A8/A9 can predict a severe or critical COVID-19 disease at an AUC of 0.827 based on the antibody array data and 0.886 based on the ELISA data (Fig. 7). At a sensitivity of 90%, a specificity of 59.6% can be reached for CRP and of 48.9% for S100A8/A9 based on the antibody array data. ROC curves for the two proteins are in good coherence for ELISA and antibody array data with the ELISA data slightly outperforming the data derived from highly multiplex antibody arrays (Fig. 7).

In order to further improve the diagnostic accuracy, especially in terms of sensitivity, potential biomarker combinations were determined via machine learning models. As linear support vector machines (linSVM) are easily interpretable and can be easily transferred into a diagnostic assay format, in our approach we focused and described models selected based on linSVMs rather than tree-based models like XGBoost.

From the many evaluated linSVM signatures, we present the ten models yielding the highest performance based on their AUC for signature lengths of two, three and four proteins, respectively, within Supplementary Table 2 and highlighted four of these models within Table 3 and Fig. 7. The identified models were compared to a combination of the top single protein biomarker candidates S100A8/A9 and CRP as well as their combination. The combination of the two biomarkers revealed an AUC of 0.830 and a specificity of 46.8% at a sensitivity of 90% and thereby a similar performance as the individual markers. For other combinations selected from the linSVM models, the overall performance and especially the sensitivity could be improved. A combination of S100A8/A9 with the protein TSP1 yielded an AUC of 0.872, thereby showing the highest AUC of all marker panels consisting of two biomarkers. If ERBB2 is added to this marker panel, the AUC is further improved to 0.898. A higher AUC of 0.913 was reached with one other panel of the three biomarkers S100A8/A9, TSP1 and IFNL1. A further addition of FINC to this marker panel increases the AUC to 0.928 with a slightly lower specificity at a sensitivity of 95% as compared to the two selected three protein biomarker panels. For a detection of a severe disease course using the four marker panels highlighted in Fig. 7, a sensitivity of 90% could be reached at specificities of 78.7%, 80.9%, 78.7% and 83.0%, respectively (Table 3), clearly outperforming all individual protein markers as well as the combination of S100A8/A9 and CRP.

**Table 3:** Specificities at given sensitivities for selected individual biomarkers and biomarker combinations.

Biomarker/Biomarker Combination	AUC	Specificity at a Sensitivity of			
		95%	90%	85%	80%
S100A8/A9	0.827	19.1%	48.9%	74.5%	76.6%
CRP	0.837	34.0%	59.6%	70.2%	76.6%
S100A8/A9 + CRP	0.830	46.8%	46.8%	74.5%	76.6%
S100A8/A9 + TSP1	0.872	70.2%	78.7%	78.7%	80.9%
S100A8/A9 + TSP1 + IFNL1	0.913	80.9%	80.9%	83.0%	83.0%
S100A8/A9 + TSP1 + ERBB2	0.898	74.5%	78.7%	85.1%	85.1%
S100A8/A9 + TSP1 + FINC + IFNL1	0.928	63.8%	83.0%	87.2%	87.2%

## Discussion

The presented study aimed to find sensitive and specific biomarkers to identify patients at high risk of developing a severe or critical course of COVID-19 within an early phase of infection and prior to the onset of severe symptoms. Antibody microarray analysis of samples, taken during the acute phase of COVID-19 infection, within the 1^st^ cohort revealed 58 proteins with an altered abundance in patients which later developed a critical or severe (CS) disease. Analysis of samples from COVID-19 patients from the 2^nd^ cohort on antibody microarrays revealed 51 proteins as biomarkers to predict a critical or severe (CS) course of a COVID-19 disease, 46 of these were higher and seven lower abundant. All samples analyzed in the 2^nd^ cohort were collected during the acute phase of infection as we specifically aimed to identify biomarkers in an early phase of infection since they may help to identify patients at risk for a severe disease course at an early disease stage and thereby allow for stratification of patients, inform optimal treatment and monitoring strategies and ultimately improve disease outcome. While a panel of laboratory indicators of severe disease have been identified, such as creatinine levels, neutrophil counts, C-reactive protein (CRP)^20^ and D-dimer levels^21^, these values represent rather unspecific markers of an increased inflammatory state and/or organ dysfunction. In employing a broader approach, the detailed description of differentially abundant protein levels in MM vs. CS patients using high-content antibody arrays as described here does provide a more complete picture.

Some of the biomarkers elevated in the acute phase of CS patients that have been identified within both analyzed cohorts have already been associated with COVID-19 disease progression in previous studies. S100A8/A9, also known as calprotectin, is a heterodimer involved in neutrophil-related inflammatory processes. Several studies have shown a correlation between S100A8/A9 and disease severity of COVID-19 with increased S100A8/A9 levels being associated with poor clinical outcomes such as significantly reduced survival time^9,22–26^. Plasma levels of fibroblast growth factor 2 (FGF2) were previously reported to be associated with severe disease and Intensive Care Unit (ICU) admission^27^. In addition, FGF2 was found to be up-regulated in lungs of patients who died from COVID-19^28^. Zhang et al. discovered two novel severe-disease-specific monocyte subsets as potential predictors and therapeutic targets for severe COVID-19^29^. Amphiregulin (AREG), a protein identified within this study, is expressed by these subsets of monocytes, which can explain increased AREG levels in CS patients. It has been shown that an increase in IL-2 can be associated with a high viral load and a severe course of the disease resulting from a hyperinflammatory state^30^. We have furthermore found that levels of insulin-like growth factor 1 receptor (IGF1R) were elevated in acute CS within the 1^st^ cohort and IGF1 levels were elevated within the 2^nd^ cohort. This is of particular interest since the IGF1/ IGF1R pathway has been implicated in immune regulation. Consistent with our observations, higher levels of IGF1R have been identified in severe and critical COVID-19 using RNAseq in a recently published pre-print^31^. Since elevated plasma levels of IGF1 and IGF1R have been observed in early acute respiratory distress syndrome (ARDS)^32^, it has been suggested that IGF1 may be a potential target in the treatment of COVID-19-related ARDS^33^.

We have identified the checkpoint inhibitor OX-2 membrane glycoprotein (OX2G)/CD200 to be less abundant in CS patients. OX2G negatively regulates the immune response in order to prevent excessive inflammation^34^. The inhibition of OX2G was described to have a positive effect on coronavirus infection by restoring interferon production and increasing virus clearance^35^. The cytokine CSF1 (macrophage colony-stimulating factor) plays an important role in the proliferation and differentiation of macrophages and monocytes and promotes the release of proinflammatory chemokines^36^. CSF1 was already identified as a biomarker involved in the main biological processes leading to severe COVID-19 manifestations and was assumed to reflect levels of lung inflammation^37^. Another biomarker identified within our study to be elevated in severe COVID-19, but not reported before, is ALCAM (Activated Leukocyte Cell Adhesion Molecule, CD166), which plays a role in transmigration of monocytes across pulmonary endothelium and in T-cell activation^38^. The ALCAM pathway has been shown to be up-regulated in patients with severe asthma^39^. Recently, Rébillard et al. suggested that ALCAM could be used as a therapeutic target in COVID-19 outcome due to its biological function and association with respiratory diseases^40^. Within the 1^st^ cohort CD28, a co-stimulatory molecule on T-cells required for T-cell activation, was increased in CS patients during the acute phase of infection. These levels decreased again during the intermediate and late stage of CS patients in the 1^st^ cohort. Decreased levels of CD28 on CD4^+^ and CD8^+^ T-cells in severe COVID-19 have previously been described and it has been suggested that this observation may be due the initial state of hyperinflammation and -activation in severe COVID-19 and a resulting state of immune exhaustion^41^, which is consistent with the observations made in our study.

The low number of patients included in the 1^st^ cohort is a limitation that needs to be acknowledged. We have analyzed a total of 53 samples from 16 different patients of which only five showed a severe or critical course of disease. Still, as outlined before also the findings from the 1^st^ cohort correlate quite well with earlier reports on biomarker candidates to predict COVID-19 progression. As age and certain comorbidities are a risk factor for a critical or severe COVID-19 disease, we controlled for these aspects in the definition of the 2^nd^ study cohort. In this cohort not all marker candidates from the first cohort could be confirmed. This might be due to the fact that some differential protein levels have been driven by individuals. Another explanation might be that these proteins are connected to age or certain comorbidities and thereby not exhibit a significance in the 2^nd^ cohort controlling for these potential cofounding factors with a case-control design.

All identified proteins might be promising biomarkers to detect individuals at a high risk for developing severe COVID-19 during an early phase of infection. Up-regulated protein levels in CS patients, observed in the acute phase, showed decreasing abundance during the intermediate and late phase for some targets when analyzing longitudinal samples from the 1^st^ cohort. Since all CS patients within this study survived COVID-19, these markers, as well as markers identified in the intermediate phase of the disease might be promising markers for therapy monitoring.

For the analysis of the 2^nd^ cohort, antibody microarrays targeting a broader range of proteins were used in order to gain more comprehensive insights into plasma protein profiles connected with the onset of severe COVID-19. A protein interaction analysis of the biomarkers to predict a critical or severe course of the disease, revealed the JAK/STAT pathway as one of the central axes in the protein interaction network. A JAK/STAT activation is frequently seen in infectious diseases and a sign for immune cell activation and proliferation. Drugs targeting the JAK/STAT pathway are currently under consideration as treatment options in COVID-19^42^. In addition, the protein interaction analysis revealed a regulation of many S100 family proteins such as S100A8/A9 and S100B in combination with HMBG1. Other mechanisms and pathways identified in the protein interaction network of the biomarker candidates are part of a systemic response to a severe viral infection. This systemic response comprises the activation of many immune cell types as well as complex regulations of growth factors and soluble inflammatory mediators, including IL15, I13R2 (HGNC: IL13RA2), CD47 and TSP1 (HGNC: THBS1).

As a response protein to inflammation, CRP was identified in the 2^nd^ cohort as one of the top individual markers Although CRP is a rather unspecific inflammation marker, it has widely been published as a prognostic biomarker for COVID-19 progression^43^, and we were able to reproduce these published data by our array platform as well as by an ELISA performed in a routine clinical diagnostics laboratory. Scotto et al. showed in correlation with our findings that MUC1 (Mucin-1) is a reliable indicator of pulmonary function, reported an association with poorer outcome and death in COVID-19 patients and therefore suggested MUC1 as a sensitive parameter to stratify the risk of severe respiratory failure and death in COVID-19 patients^44^. As in our study, enhanced expression of CCL3^45,46^ and ANGP2^47^ were also described as predictive factors in differentiating COVID-19 patients and determining severity of disease.

Individual biomarker candidates with the highest log-fold change in the 2^nd^ cohort exhibited also the highest diagnostic accuracy with an AUC of 0.83. Still, this performance might not be sensitive enough, especially in a larger study population with a higher number of mild and moderate cases. Therefore, machine learning approaches were applied to select ideal marker combinations for improved AUC as well as sensitivity. To this end, linear as well as decision tree approaches were tested. While tree-based approaches based on XGBoost had a slightly better performance, we decided to focus on linear approaches based on linSVM, as linear models can be more easily reproduced for the setup of a multiplex assay aiming at in vitro diagnostic certification.

Due to the larger sample size of the 2^nd^ cohort and the divergence in selection of samples in the two studies – matched mild/moderate and severe/critical cases in respect to age, gender, and days after onset of symptoms only in the 2^nd^ cohort -, the 2^nd^ cohort was used for the linSVM approach to select biomarker combinations discriminating between patients with CS and MM disease courses at a higher accuracy than individual protein biomarkers. A combination of the four proteins S100A8/9, FINC, IFNL1 and TSP1 (AUC = 0.928) as well as of the three proteins S100A8/A9, TSP1 and ERBB2 (AUC = 0.898) but also of the two protein biomarkers S100A8/A9 and TSP1 (AUC = 0.872) outperformed individual biomarkers such as S100A8/A9 (AUC: 0.827) or CRP (AUC: 0.837) clearly. FINC and TSP1 levels were not measured within the 1^st^ cohort but were significantly higher in MM patients as compared to CS patients within the 2^nd^ cohort. From the proteins with specific importance in the biomarker signatures identified by machine learning, FINC (Fibronectin) has not yet been published as a biomarker for severe COVID-19, however it has been identified in inflammation and sepsis as a negative acute-phase protein, with a low level of FINC indicating a poor prognosis for a patient^48^. For Thrombospondin-1 (TSP1) we did not identify any report as a COVID-19 disease severity biomarker, however TSP1 protein levels were shown to be up-regulated in the serum of infected asymptomatic individuals as compared to negative individuals^49^. In lung cancer patients, lower TSP1 levels are associated with a higher lymph node involvement in lung cancer^50^. IFNL1 levels were slightly higher in MM patients as compared to CS patients within the 2^nd^ cohort. Although differences in IFNL1 were not significant as an individual biomarker, IFNL1 was selected by the linear SVM approach selecting combinations of biomarkers to improve sensitivity and specificity as compared to a single biomarker. Lambda IFNs such as INFL1 are produced as a response to a viral infection and act in mucosal barriers, e.g. in the respiratory tract. The concentrations of lambda IFNs induced in the serum of non-ICU COVID-19 patients were shown to be higher than in ICU COVID-19 patients^51^. Additionally, it was shown that IFNL1 restricts SARS-CoV-2 replication in cellular models of viral infection when administered preventively and/or therapeutically^52^. In MERS-CoV, lambda IFN greatly limits viral replication, and may be a key cytokine for better therapeutic outcomes against MERS-CoV infection in the respiratory tract^53^.

All machine learning approaches were performed using data based on log-ratios of sample and reference as derived from the antibody array platform. Such multiplex antibody array data proved very valuable for machine learning, as, contrary to many other platforms, there is no issue of missing data.

As demonstrated for the two individual top biomarker candidates S100A8/A9 and CRP, the array data could be very well correlated (*r* > 0.9) and reproduced by commercial ELISA. In a direct comparison by a side-to-side ROC analysis of array and ELISA data, the ROC curves for S100A8/9 and CRP originating from the ELISA data slightly outperformed the antibody array data. These differences are likely a result of a combination of factors, such as a more complex experimental layout (reference-based), different laboratory procedures, in particular adjustment of bulk protein concentrations for plasma samples, and data normalization on the side of the array data.

Nonetheless, these findings indicate a very good transferability of the results of this study to routine platforms for protein analysis with a low risk of false-positive findings. This direct transferability of results to other immune based platforms used in routine diagnostics such as ELISA is a major advantage of antibody array-based proteomics. Additional advantages of the antibody microarray platform include the highly simultaneous screening of several hundred (1^st^ cohort) and more than a thousand (2^nd^ cohort) proteins from less than fifty microliters of plasma sample in a comparably high throughput, and sensitivity levels enabling a robust cytokine profiling from complex samples without the need of an intensive sample depletion and fractionation as required in mass spectrometry-based approaches.

Based on the Sciomics antibody microarray platform we were able to identify plasma protein biomarkers to predict a severe COVID-19 disease, selected protein biomarker combinations with an accuracy suitable for a clinical application and validated two of the individual protein biomarkers by commercial assays. The identified proteins are promising biomarkers to predict disease severity during an early phase of SARS-CoV-2 infection. The evidence that is available to date suggests that these markers are connected to mechanisms involved in disease progression to severe COVID-19 as well as in other infectious disease models. Individually or collectively, these markers can identify patients at high risk of developing a severe and critical course of disease and can inform treatment and clinical care choices.

### Reporting summary

Further information on research design is available in the Nature Portfolio Reporting Summary linked to this article.

## Supplementary information

Description of Additional Supplementary Files

Supplementary Material

Supplementary Data 1

Supplementary Data 2

Supplementary Data 3

Reporting Summary

## Data Availability

The authors declare that all logFC data, generated during the microarray analysis are available within the paper and its supplementary information files (Supplementary Data 1 and Supplementary Data 2). Raw red and green signal intensities and the resulting normalised M-values of both cohorts are deposited at ArrayExpress (1st cohort: E-MTAB-12779 Discovery and systematic assessment of early biomarkers that predict progression to severe COVID-19 disease - 1st cohort; 2nd cohort: E-MTAB-12777 Discovery and systematic assessment of early biomarkers that predict progression to severe COVID-19 disease - 2nd cohort). Data for reproducing the figures is available within the supplementary data 3.

## Code availability

Microarray raw image files were processed using GenePix Pro 6.0 and statistical analysis was performed using the limma package (3.42.2) in R (3.6.3) as outlined in detail in a publication describing the data analysis routines for antibody microarray analyses^16^. Machine learning procedures were performed using the scikit-learn package (0.24.2) in python (3.8.10) according to the methodology described in the Materials & Methods section. The code was published and can be accessed via the following DOI: 10.5281/zenodo.7759895^18^.
