# Supplementary material for: Discovery and systematic assessment of early biomarkers that predict progression to severe COVID-19 disease
Source: Commun Med (Lond). 2023 Apr 12;3:51. doi: 10.1038/s43856-023-00283-z (PMC10089829; doi:10.1038/s43856-023-00283-z)
Supplement: Supplementary file 1 — Description of Additional Supplementary Files [file 43856_2023_283_MOESM1_ESM.pdf]

Description of Additional Supplementary Files

File Name: Supplementary Data 1

Description: All analyzed proteins with their respective abundance in CS and MM patients in the three disease phases within the 1st cohort are listed in Supplementary Data 1. For each protein, the Uniprot Entry-Name and ID is listed together with the logFC and adjusted p values.

File Name: Supplementary Data 2

Description: All analyzed proteins with their respective abundance in CS and MM patients within the 2nd cohort are listed in Supplementary Data 2. For each protein, the Uniprot Entry-Name and ID is listed together with the logFC and adjusted p values.

File Name: Supplementary Data 3

Description: Source data underlying the graphs and charts presented in the main figures.

Figure 1: Patient disease severity, sample extraction time points, age, sex.
Figure 2: logFCs, logarithmized adjusted p values, protein labels and colors for each volcano plot.
Figure 3: Sample Points: x_positions and intensity values for each respective antibody stripchart.
Statistics - mean, stdev, linear model fit: means, standard deviations and linear model coefficients (incorporating age, sex, and comorbidities) for each respective antibody stripchart.
Figure 4: logFCs, logarithmized adjusted p values, protein labels and colors for the volcano plot.
Figure 5: Sample Points: x_positions and intensity values for each respective antibody stripchart.
Statistics - mean, stdev, linear model fit: means, standard deviations and linear model coefficients (incorporating age, sex, and comorbidities) for each respective antibody stripchart.
