# Supplementary material for: Discovery and systematic assessment of early biomarkers that predict progression to severe COVID-19 disease
Source: Commun Med (Lond). 2023 Apr 12;3:51. doi: 10.1038/s43856-023-00283-z (PMC10089829; doi:10.1038/s43856-023-00283-z)
Supplement: Supplementary file 2 — Supplementary Material [file 43856_2023_283_MOESM2_ESM.pdf]

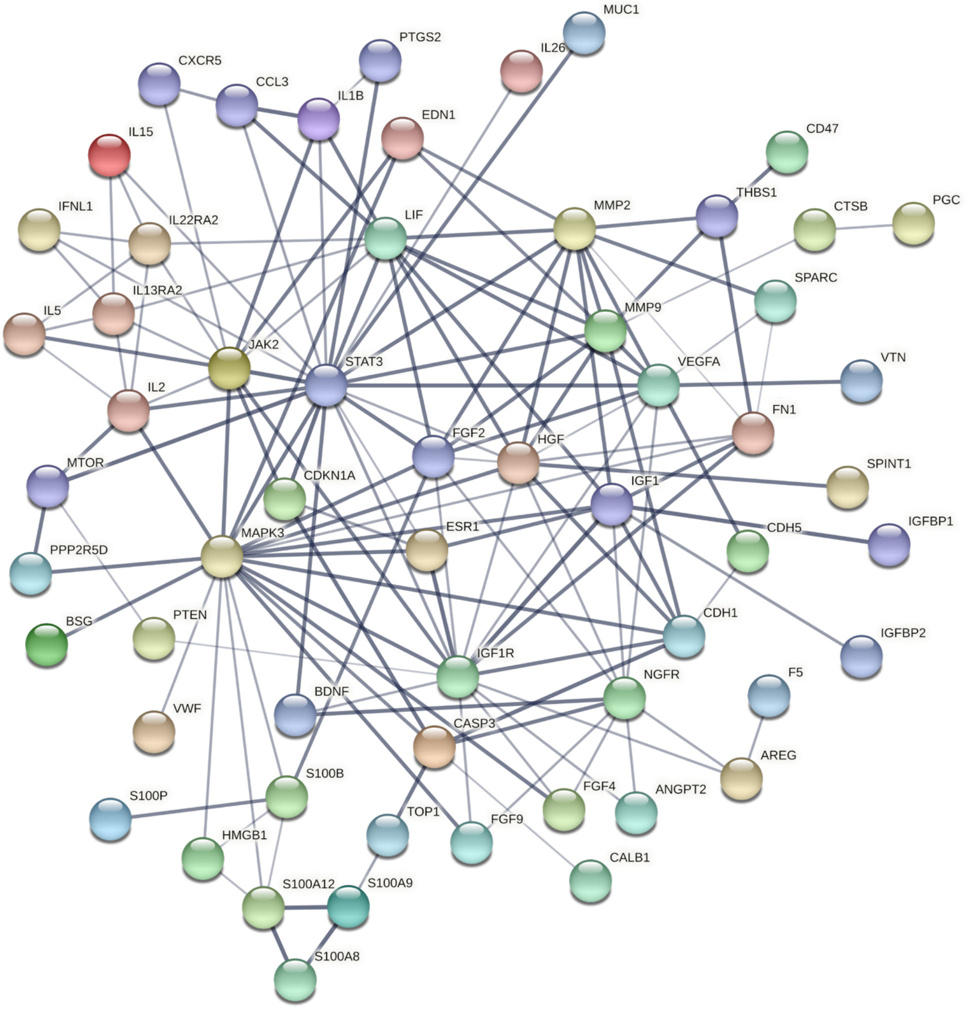


**Supplementary Figure 1: Protein-protein interaction graph of differential proteins of the 2nd cohort generated using the STRING database** (http://string-db.org). Information from experiments, databases, co-expression, neighborhood, gene fusion and co-occurrence were included as interaction sources, and interactions with a confidence score of above 0.4 were displayed.

***Supplementary Table 1: Top 20 marker pre-selection for machine learning.*** *All 11 markers which displayed discriminative power based on logFC and p values obtained from the linear models of both cohorts were preselected. For markers which featured the same regulation direction, i.e. the logFC sign, across both cohorts, the potential impact for linSVM models was gauged by training preliminary models in a LOO manner and summing up the resulting absolute linear coefficients over all models. The top 10 markers according to this coefficient sum were included into the pre-selection of markers for the machine learning signature evaluation.*

| Differential in both cohorts | | | | | |
|---|---|---|---|---|---|
| Target | Uniprot ID | 1st cohort | | 2nd cohort | |
| | | logFC | adj.p-val | logFC | adj.p-val |
| S100A8/A9 | P05109/ P06702 | 1.59 | 0,013 | 1.71 | $6.4 \times 10^{-14}$ |
| FGF2 | P09038 | 2.43 | $5.8 \times 10^{-6}$ | 1.31 | $1.7 \times 10^{-5}$ |
| SLAF1 | Q13291 | 2.41 | $3.7 \times 10^{-4}$ | 1.79 | $2.1 \times 10^{-5}$ |
| CD47** | Q08722 | 1.75 | $5.3 \times 10^{-4}$ | 1.25 | $2.4 \times 10^{-4}$ |
| CXCR5 | P32302 | 2.94 | $3.7 \times 10^{-4}$ | 1.25 | $9.6 \times 10^{-4}$ |
| I13R2 | Q14627 | 1.64 | $3.5 \times 10^{-6}$ | 0.99 | $2.7 \times 10^{-3}$ |
| CD81 | P01889 | 1.54 | 0,038 | 0.82 | $2.7 \times 10^{-3}$ |
| AREG | P31997 | 1.84 | $8.6 \times 10^{-3}$ | 1.30 | $3.5 \times 10^{-3}$ |
| TNR16 | P08138 | 2.40 | $1.0 \times 10^{-4}$ | 1.09 | $8.9 \times 10^{-3}$ |
| IL2 | P60568 | 2.72 | $3.4 \times 10^{-4}$ | 1.03 | 0.010 |
| BTLA | Q7Z6A9 | 1.27* | $1.6 \times 10^{-3}$* | 1.15 | 0.016 |

| High potential impact gauged from preliminary analysis | | |
|---|---|---|
| Target | Uniprot ID | linSVM coefficient score |
| TSP1 | P07996 | 21.20 |
| CD38 | P01730 | 21.04 |
| FINC | P02751 | 17.67 |
| IFNL1 | Q8IU54 | 17.22 |
| ERBB2 | P04626 | 16.66 |
| CD47 | Q08722 | 15.74 |
| MMP9 | P14780 | 15.25 |
| PTEN | P60484 | 14.67 |
| ISK1 | P00995 | 13.76 |
| IL15 | P40933 | 13.48 |

*BTLA logFC and p-values (p-val.) for the 1st cohort are from the intermediate phase of infection
**two different antibodies were included in linSVM

*Supplementary Table 2: Specificities at given sensitivities for the top 10 biomarker combinations for signature lengths of two, three and four proteins yielding the highest performance based on their AUC.*

| | Biomarker Combination | | | | AUC | Specificity at a Sensitivity of | | | |
|---|---|---|---|---|---|---|---|---|---|
| | Marker1 | Marker2 | Marker3 | Marker4 | | 95% | 90% | 85% | 80% |
| signature lengths of two proteins | S10A8/9 | TSP1 | | | 0,872 | 70,2 | 78,7 | 78,7 | 80,9 |
| | S10A8/9 | IFNL1 | | | 0,871 | 48,9 | 68,1 | 80,9 | 80,9 |
| | S10A8/9 | ERBB2 | | | 0,847 | 44,7 | 57,4 | 83,0 | 83,0 |
| | S10A8/9 | FINC | | | 0,845 | 21,3 | 61,7 | 72,3 | 78,7 |
| | S10A8/9 | IL15 | | | 0,831 | 10,6 | 38,3 | 70,2 | 83,0 |
| | S10A8/9 | SLAF1 | | | 0,821 | 19,1 | 48,9 | 74,5 | 76,6 |
| | S10A8/9 | CD81 | | | 0,818 | 17,0 | 36,2 | 63,8 | 78,7 |
| | S10A8/9 | IL2 | | | 0,815 | 21,3 | 40,4 | 72,3 | 76,6 |
| | S10A8/9 | CD38 | | | 0,814 | 10,6 | 46,8 | 66,0 | 66,0 |
| | S10A8/9 | I13R2 | | | 0,810 | 17,0 | 51,1 | 70,2 | 70,2 |
| signature lengths of three proteins | S10A8/9 | TSP1 | IFNL1 | | 0,913 | 80,9 | 80,9 | 83,0 | 83,0 |
| | S10A8/9 | TSP1 | ERBB2 | | 0,898 | 74,5 | 78,7 | 85,1 | 85,1 |
| | S10A8/9 | FINC | IFNL1 | | 0,896 | 25,5 | 68,1 | 80,9 | 89,4 |
| | TSP1 | FINC | IFNL1 | | 0,878 | 40,4 | 42,6 | 74,5 | 89,4 |
| | S10A8/9 | TSP1 | FINC | | 0,876 | 44,7 | 74,5 | 74,5 | 76,6 |
| | S10A8/9 | IFNL1 | IL15 | | 0,875 | 31,9 | 66,0 | 83,0 | 83,0 |
| | S10A8/9 | TSP1 | CD38 | | 0,874 | 66,0 | 76,6 | 80,9 | 80,9 |
| | S10A8/9 | IFNL1 | ERBB2 | | 0,874 | 40,4 | 70,2 | 78,7 | 80,9 |
| | S10A8/9 | CD81 | IFNL1 | | 0,872 | 44,7 | 61,7 | 80,9 | 80,9 |
| | S10A8/9 | CD81 | TSP1 | | 0,871 | 68,1 | 74,5 | 76,6 | 80,9 |
| signature lengths of four proteins | S10A8/9 | TSP1 | FINC | IFNL1 | 0,928 | 63,8 | 83,0 | 87,2 | 87,2 |
| | S10A8/9 | TSP1 | CD38 | IFNL1 | 0,922 | 72,3 | 83,0 | 85,1 | 85,1 |
| | S10A8/9 | IL2 | TSP1 | IFNL1 | 0,919 | 78,7 | 80,9 | 85,1 | 85,1 |
| | S10A8/9 | FINC | IFNL1 | IL15 | 0,917 | 40,4 | 80,9 | 80,9 | 91,5 |
| | S10A8/9 | TSP1 | IFNL1 | ERBB2 | 0,916 | 76,6 | 80,9 | 83,0 | 83,0 |
| | S10A8/9 | CD47 | TSP1 | IFNL1 | 0,916 | 78,7 | 78,7 | 83,0 | 87,2 |
| | S10A8/9 | TSP1 | IFNL1 | CD47 | 0,916 | 78,7 | 78,7 | 83,0 | 87,2 |
| | S10A8/9 | TSP1 | IFNL1 | ISK1 | 0,916 | 74,5 | 80,9 | 83,0 | 85,1 |
| | S10A8/9 | AREG | TSP1 | IFNL1 | 0,912 | 78,7 | 80,9 | 80,9 | 83,0 |
| | S10A8/9 | I13R2 | TSP1 | IFNL1 | 0,911 | 80,9 | 80,9 | 80,9 | 83,0 |

*Color code:*
*dark green: specificity > 85%; light green: specificity between 80% and 85%; yellow: specificity between 70% and 80%; light red: specificity between 60% and 70%; dark red: specificity < 60%*
