# Supplementary material for: Discovery and systematic assessment of early biomarkers that predict progression to severe COVID-19 disease
Source: Commun Med (Lond). 2023 Apr 12;3:51. doi: 10.1038/s43856-023-00283-z (PMC10089829; doi:10.1038/s43856-023-00283-z)
Supplement: Supplementary file 6 — Reporting Summary [file 43856_2023_283_MOESM6_ESM.pdf]

nature research

Corresponding author(s): Dr Christoph Schröder

Last updated by author(s): Mar 28, 2023

Reporting Summary

Nature Research wishes to improve the reproducibility of the work that we publish. This form provides structure for consistency and transparency in reporting. For further information on Nature Research policies, see our Editorial Policies and the Editorial Policy Checklist.

Statistics

For all statistical analyses, confirm that the following items are present in the figure legend, table legend, main text, or Methods section.

n/a	Confirmed	
☐	☒	The exact sample size (n) for each experimental group/condition, given as a discrete number and unit of measurement
☐	☒	A statement on whether measurements were taken from distinct samples or whether the same sample was measured repeatedly
☐	☒	The statistical test(s) used AND whether they are one- or two-sided<br>*Only common tests should be described solely by name; describe more complex techniques in the Methods section.*
☐	☒	A description of all covariates tested
☐	☒	A description of any assumptions or corrections, such as tests of normality and adjustment for multiple comparisons
☐	☒	A full description of the statistical parameters including central tendency (e.g. means) or other basic estimates (e.g. regression coefficient) AND variation (e.g. standard deviation) or associated estimates of uncertainty (e.g. confidence intervals)
☐	☒	For null hypothesis testing, the test statistic (e.g. F, t, r) with confidence intervals, effect sizes, degrees of freedom and P value noted<br>*Give P values as exact values whenever suitable.*
☒	☐	For Bayesian analysis, information on the choice of priors and Markov chain Monte Carlo settings
☒	☐	For hierarchical and complex designs, identification of the appropriate level for tests and full reporting of outcomes
☐	☒	Estimates of effect sizes (e.g. Cohen's d, Pearson's r), indicating how they were calculated

Our web collection on statistics for biologists contains articles on many of the points above.

Software and code

Policy information about availability of computer code

Data collection	GenePix Pro 6.0
Data analysis	R 3.6.3, limma 3.42.2, python 3.8.10, sklearn 0.24.2, pandas 1.3.0

For manuscripts utilizing custom algorithms or software that are central to the research but not yet described in published literature, software must be made available to editors and reviewers. We strongly encourage code deposition in a community repository (e.g. GitHub). See the Nature Research guidelines for submitting code & software for further information.

Data

Policy information about availability of data

All manuscripts must include a data availability statement. This statement should provide the following information, where applicable:

- Accession codes, unique identifiers, or web links for publicly available datasets
- A list of figures that have associated raw data
- A description of any restrictions on data availability

The authors declare that all logFC data, generated during the microarray analysis are available within the paper and its supplementary information files. Raw red and green signal intensities and the resulting normalised M-values of both cohorts are deposited at ArrayExpress (1st cohort: E-MTAB-12779 Discovery and systematic assessment of early biomarkers that predict progression to severe COVID-19 disease - 1st cohort; 2nd cohort: E-MTAB-12777 Discovery and systematic assessment of early biomarkers that predict progression to severe COVID-19 disease - 2nd cohort). Data for reproducing the figures is available within the supplementary data 3.

Field-specific reporting

Please select the one below that is the best fit for your research. If you are not sure, read the appropriate sections before making your selection.

☒ Life sciences ☐ Behavioural & social sciences ☐ Ecological, evolutionary & environmental sciences

For a reference copy of the document with all sections, see nature.com/documents/nr-reporting-summary-flat.pdf

Life sciences study design

All studies must disclose on these points even when the disclosure is negative.

Sample size	First cohort: We analyzed 53 plasma samples collected longitudinally from 16 COVID-19 patients.<br>Second cohort: We analyzed 94 plasma samples from COVID-19 patients during the acute phase of disease.
Data exclusions	No data were excluded from the analysis.
Replication	Each antibody was measured in four technical replicates.
Randomization	Samples were randomized for each laboratory step.
Blinding	Each sample was assigned to an internal ID. During analysis in the lab no assigment to study relevant data was possible.

Reporting for specific materials, systems and methods

We require information from authors about some types of materials, experimental systems and methods used in many studies. Here, indicate whether each material, system or method listed is relevant to your study. If you are not sure if a list item applies to your research, read the appropriate section before selecting a response.

Materials & experimental systems

n/a	Involved in the study	
☐	☒	Antibodies
☒	☐	Eukaryotic cell lines
☒	☐	Palaeontology and archaeology
☒	☐	Animals and other organisms
☐	☒	Human research participants
☒	☐	Clinical data
☒	☐	Dual use research of concern

Methods

n/a	Involved in the study	
☒	☐	ChIP-seq
☒	☐	Flow cytometry
☒	☐	MRI-based neuroimaging

Antibodies

Antibodies used	For the measurement of CRP a clinically validated assay was used, for the measurement of S100A8/A9 a commercial Duoset Elisa by R&D.
Validation	n.a.

Human research participants

Policy information about studies involving human research participants

Population characteristics	First cohort:<br>We analyzed 53 plasma samples collected longitudinally from 16 COVID-19 patients. The cohort included eight men and women, respectively, aged between 23 and 85 years (mean: 47 years, SD: 18 years). We decided to divide the disease course into three periods based on days since the onset of first symptoms: an acute (< 10 days) an intermediate (between 10 and 21 days) and a convalescent/late stage (> 21 days), for which we included 18, 19 and 16 samples, respectively. In all individuals, the day of first symptom onset was between February 25th and April 30th, 2020, and the cohort, therefore, mirrors an early time of the pandemic, when specific therapies against COVID-19 had not been established. Immuno-modulating/suppressive conditions and therapies were recorded for all participants to be able to assess whether these conditions/treatments influenced our findings. Of the 16 individuals, 2 were pregnant (patients e and h), one individual received 7.5 mg prednisolone (patient g), another one 6 mg dexamethasone daily due to underlying comorbidities (patient o) and one had received tocilizumab as a compassionate use treatment of COVID-19 (j). The remaining 11/16 patients did not have any immuno-modulating or -suppressive condition and did not receive any immunosuppressive therapy.<br>Second cohort:<br>We analyzed 94 plasma samples from COVID-19 patients during the acute phase of disease (< 10 days after the onset of first

	symptoms). From these patients, 47 patients had a critical or severe course of disease. Additionally, 47 age and sex matched samples from 47 patients with a mild to moderate disease were analyzed. All patients survived the infection and did not receive COVID-19 specific medication prior to sample collection.
Recruitment	After obtaining written informed consent, whole blood samples from SARS-CoV-2 infected individuals, who had been diagnosed by RT-PCR using a nasopharyngeal swab, were collected at University Medical Center Hamburg-Eppendorf (1st cohort) and Department of Gastroenterology and Infectious Diseases of University Hospital Heidelberg (2nd cohort) at different timepoints after diagnosis.
Ethics oversight	Both studies were conducted according to the ethical requirements established by the Declaration of Helsinki. The 1st study was approved by local Ethics Committee of the Hamburg Medical Association (ethic consent number PV7298) while the 2nd study was approved by the local Ethics Committee of the Medical Faculty of Heidelberg University Hospital (ethic consent number S-148/2020).

Note that full information on the approval of the study protocol must also be provided in the manuscript.
